# Reproducible Construction of Surface Tension-Mediated Honeycomb Concave Microwell Arrays for Engineering of 3D Microtissues with Minimal Cell Loss

**DOI:** 10.1371/journal.pone.0161026

**Published:** 2016-08-11

**Authors:** GeonHui Lee, JaeSeo Lee, HyunJik Oh, SangHoon Lee

**Affiliations:** 1 KU-KIST Graduate School of Converging Science and Technology, Korea University, Republic of Korea; 2 School of Biomedical Engineering, College of Health Science, Korea University, Seoul, Republic of Korea; 3 Department of Bio-convergence Engineering, College of Health Science, Korea University, Seoul, Republic of Korea; 4 MicroFIT R&BD Institute, Dunchon-daero, Jungwon-gu, Gyeonggi-do, Republic of Korea; Texas A&M University College Station, UNITED STATES

## Abstract

The creation of engineered 3D microtissues has attracted prodigious interest because of the fact that this microtissue structure is able to mimic in vivo environments. Such microtissues can be applied extensively in the fields of regenerative medicine and tissue engineering, as well as in drug and toxicity screening. Here, we develop a novel method of fabricating a large number of dense honeycomb concave microwells via surface tension-mediated self-construction. More specifically, in order to control the curvature and shape of the concavity in a precise and reproducible manner, a custom-made jig system was designed and fabricated. By applying a pre-set force using the jig system, the shape of the honeycomb concave well was precisely and uniformly controlled, despite the fact that wells were densely packed. The thin wall between the honeycomb wells enables the minimization of cell loss during the cell-seeding process. To evaluate the performance of the honeycomb microwell array, rat hepatocytes were seeded, and spheroids were successfully formed with uniform shape and size. Liver-specific functions such as albumin secretion and cytochrome P450 were subsequently analyzed. The proposed method of fabricating honeycomb concave wells is cost-effective, simple, and reproducible. The honeycomb well array can produce multiple spheroids with minimal cell loss, and can lead to significant contributions in tissue engineering and organ regeneration.

## Introduction

Multicellular organisms consist of cells organized in three-dimensional (3D) environment in which cells continuously interact with neighboring cells. And cells cultured in 3D environment shows more in vivo cellular behaviors than that of 2D environment such as differentiation, proliferation, and gene expression [1]. Thus, culturing of cells under the 3D environment maintaining cell-cell interaction has been one of biggest issues. The engineered 3D microtissues cultured under this 3D environment have been broadly used for the regeneration of organ function, disease or pharmacokinetic model for the screening and stem cell researches [2–5]. In order to expand the application of such microtissues, production of tissues with uniform size and shape under the controllable microenvironment is critical. For example, the engineered 3D microtissues have been implanted in the body for the organ function regeneration, and the size uniformity of microtissue is critical. Jun. et al reported that the viability and function of intact islets is affected by the size, and they have shown that engineered 3D islets with uniform size and shape play a critical role in regulating the glucose levels of diabetic rats [6]. Similarly, engineered uniform-size 3D liver tissues with vascular structure enabled enhanced viability (> 2 months) and liver function; moreover, a rat liver with a 90% hepatectomy was successfully regenerated after implanting these 3D live tissues [7]. Therefore, well-organized 3D cell culture methods capable of producing a large quantity of 3D microtissues with a uniform size and shape represent significant forces in stem cell research and organ regeneration [8]. Owing to these considerations, the creation of well-defined 3D microtissues has been a considerable challenge, as well as a topic of keen research interest.

Several 3D cell culture methods have been developed for engineering 3D microtissues, including hanging drop [9–11], modified surfaces [12–14], rotating bioreactors, and magnetic levitation techniques [15–18]. These techniques enable cells to form 3D microstructure using surface tension, chemical adhesion, and physical forces. Despite their excellent contributions to the creation of 3D microtissues, these methods are limited in their ability to reproduce 3D cell-based tissues of uniform size; additional problems include the regulation of size and shape, as well as the control of the microenvironment surrounding the aggregated cells.

Recent progress in microfabrication technology has facilitated the development of diverse microscale platforms for engineering 3D tissues using simple and cost-effective processes that employ a wide range of materials. The representative microplatform is the microwell array, and various types of cells–including stem cells, cancer cells, liver cells, pancreas cells, and brain cells–have been used to create different tissue models [19–29]. Once seeded in the microwell arrays, cells spontaneously self-aggregate, ultimately forming 3D spheroids. Cylindrical and concave-arrayed microwells have been popular tools for producing spheroids of uniform size and shape [24, 30]. Among these microplatform-based 3D aggregation methods, concave structures with spheroidal contours have attracted considerable attention; that is because this structure is advantageous for the production of uniformly sized and shaped spheroids [24, 31–35]. Nevertheless, although achieving a concave structure is important, fabricating a curved bottom using conventional lithography processes is challenging.

Another critical issue involves the seeding of cells with minimum cell loss, as certain cells are expensive and difficult to access. Furthermore, obtaining the maximum production of 3D tissues within a limited area is important from the perspective of cost and labor. For this purpose, the thickness of the wall between microwells should be small, and the position of arrayed microwells should be as compact as possible. However, it is more challenging to fabricate such arrayed microwell structures with concave bottoms.

We have previously developed two simple methods for fabricating concave bottoms. According to one method, a thin elastic membrane is deflected by applying vacuum pressure, and the deflected membrane is replicated using UV-curable material [24, 36, 37]. This method is simple and does not use a lithography process; however, it is difficult to prepare a thin membrane and precisely control the force with this technique. A second method developed by Jeong et al. uses the surface tension of viscose solution to produce concave microwells [30]. According to this method, the concave bottom is created by pouring a poly(dimethylsiloxane) (PDMS) prepolymer onto a cylindrical microwell array (fabricated by a soft lithography process) and manually raking the prepolymer out with a flat plate, resulting in the meniscus-induced formation of concave structures in each well [30, 38]. Despite the simplicity of this process, it is difficult to control the shape of the concave microwells, and the process itself is not reproducible because of the manual element involved. Therefore, a new approach that is able to address the limits of both methods is urgently required.

In this paper, we develop a custom-made machine for fabricating the homogeneous shape of the concave microwell by applying uniform force and raking the prepolymer with uniform speed. The force on the PDMS microwells is automatically controllable, and the curvature of the concave structure can be modulated by varying the amount of force. Moreover, the uniform speed applied by an automated linear stage enhances the uniformity of the well shape. With the proposed device, honeycomb-structured concave microwells with thin walls were fabricated in order to minimize cell loss by decreasing the dead area of walls between microwells. As the thickness of walls decreases, most seeded cells fall into honeycomb concave microwells because the top area of the wall is narrow, thus making it possible to effectively seed cells with minimal cell loss and without a laborious cell-washing process. This honeycomb structure therefore enables the increase of spheroid density; additionally, the consumption of media and diverse biomolecules could be reduced. The performance of this system was verified by preparing and seeding primary rat hepatocytes and demonstrating the successful formation of spheroids. The creation of well-defined spheroids with the desired size and properties (e.g., co-cultured cell populations) is acutely important for studies concerning liver physiology, drug efficacy and the screening of toxicity, and the regeneration of liver function [39–42].

## Materials & Methods

### Fabrication of honeycomb concave microwell arrays using a jig system

**Fig 1:**
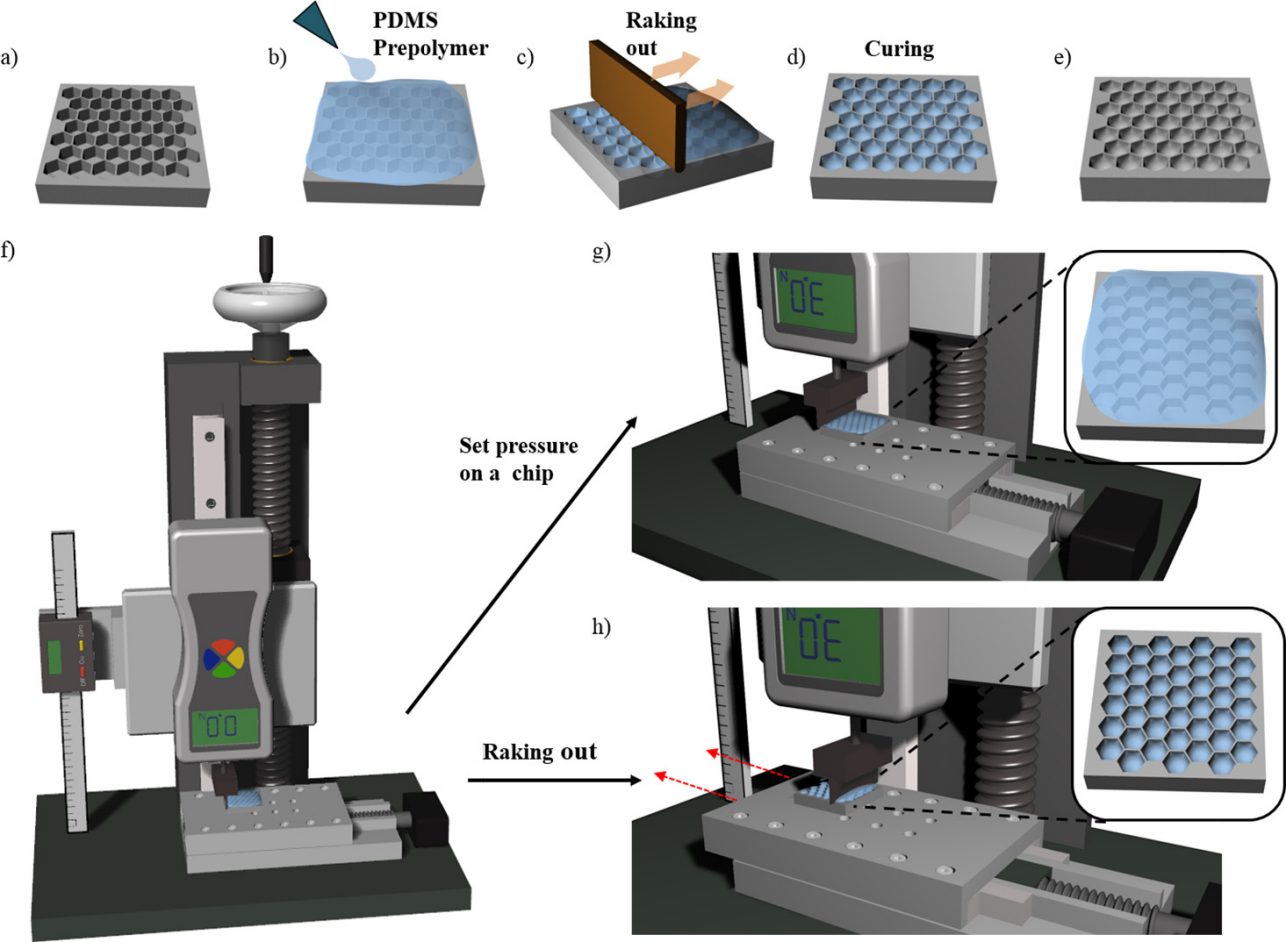
Fabrication of a honeycomb concave microwell chip. (a) Poly(dimethylsiloxane) (PDMS) honeycomb structures are fabricated using standard lithography. (b) A PDMS prepolymer is poured onto a PDMS base chip to fill the microwell, (c) and is then raked out using a flat plate with trimmed edges. (d, e) Surface tension-mediated honeycomb concave microwells are thermally cured on a hot plate. (f) A custom-designed jig system is used to fabricate honeycomb concave microwells. (g) Constant force is applied to the chip on the linear stage. (h) The PDMS prepolymer is raked out from the chip as the motorized linear stage moves in one direction.

Arrayed PDMS honeycomb microwells with flat bottoms and various diameters (300–1000 μm) were fabricated using a conventional soft lithography process [38, 43]. The shape of the well bottom was controlled by the meniscus created by the surface tension of the PDMS prepolymer, which converted the shape from flat to concave. The process for fabricating the concave bottom is illustrated in Fig 1A. We first poured a PDMS prepolymer (mixture of 10:1 silicon elastomer [Sylgard 184] and curing agent) onto the honeycomb structure and filled all the microwells; air bubbles were removed by applying a vacuum pressure of approximately −100 kPa (Fig 1B) for 30 min. We then positioned the chip on the linear stage of a jig system, as shown in Fig 1F and 1G. The PDMS prepolymer on the microwell array was raked out as the linear stage moved at a speed of 5 mm/s (Fig 1C and 1H). After raking out the prepolymer, diversely shaped concave meniscuses were self-organized in the honeycomb microwells through the surface tension of the PDMS. The prepolymer in honeycomb microwells was thermally cured on a hot plate at 80°C for 1 h (Fig 1D and 1E). The PDMS honeycomb concave microwells were replicated by pouring SU-8 (MicroChem, USA) onto the microwells, followed by UV exposure and thermal curing. The PDMS concave microwells were fabricated using the SU-8 master mold. Two types of concave microwells–circular and honeycomb–were prepared, and we then compared the formation of multiple spheroids on the two types of microwells.

### Design and fabrication of custom-made jig system

A custom-made jig system was developed for fabricating homogeneous concave microwells using the two-step process of application of constant force and raking to a prepolymer-filled chip (Fig 1F). The jig system consists of a ball screw, a linear motion (LM) guide block, a digital vernier caliper, a digital force gauge, and a motorized linear stage. The ball screw was assembled onto the LM guide block to control z-axis linear motion. The digital vernier caliper and digital force gauge were assembled onto the LM guide block to measure z-axis movement and the force on the chip. The PDMS prepolymer was raked out at constant velocity using a motorized linear stage attached to the bottom of the jig system.

### Isolation and culture of primary hepatocytes

SD (Sprague Dawwley) male rats (8 weeks) (KOATECH, Republic of Korea) were sacrificed by CO2 inhalation for the isolation of hepatocytes and hepatic stellate cells. Primary hepatocytes were isolated from 8-week-old, male Sprague-Dawley rats (DBL, Seoul, Republic of Korea) using a two-step collagenase perfusion procedure [44, 45]. Isolated hepatocytes were cultured in high-glucose Dulbecco’s Modified Eagle’s Medium (DMEM) supplemented with 20 mM 4-(2-hydroxyethyl)-1-piperazineethanesulfonic acid (HEPES) (Sigma Aldrich), 25 mM NaHCO_3_, 30 mg/mL L-proline, 10% fetal bovine serum (FBS), 25 U/mL penicillin, 25 mg/mL streptomycin, 10 mg/mL gentamicin, 10 ng/mL epidermal growth factor (EGF), 50 ng/mL insulin, 10^−4^ M dexamethasone, 10 mM nicotinamide, and 100 mM L-ascorbic acid. Hepatocytes were cultured in a humidified incubator at 37°C with 5% CO_2_. All experimental procedures carried out in strict accordance with the guidelines of the Institutional Animal Care and Use Committee of Korea University (KUIACUC). The protocol was approved by the KUIACUC (Permit Number: KUIACUC-152-2015-111). All efforts were made to minimize suffering.

### Hepatocyte seeding and spheroid formation

Hepatocytes were seeded and spheroids were self-aggregated in PDMS-based concave microwells. Concave microwell array chips were sterilized by being autoclaved and dried in an oven for 1 h. In order to prevent cell attachment, we coated the concave PDMS chip with 3% (w/v) bovine serum albumin (BSA) overnight in an incubator, after which the chip was rinsed with phosphate-buffered solution (PBS). A suspension of primary hepatocytes (400 μL with 4 × 10^5^ cells/mL) was directly seeded onto the concave microwells. The cells were evenly docked within the concave microwells.

### Measurements of cell loss ratio

Cell loss during the seeding process was measured by counting the cells in the external region of the microwells. After seeding hepatocytes in each group of microwell, we gathered the undocked cells by pipetting and counting the number of cells retrieved. Cell loss ratios were calculated as the number of cells retrieved from pipetting divided by the initial number of seeded cells, and the ratios of each group (i.e., the circular and honeycomb arrays) were compared. After 7 days of culturing, we measured the number of cells in each spheroid of each group. We counted the number of spheroids and treated them with a 1:1 mixture of TrypLE Express (gibco) and 1X HBSS (gibco) for 5 min at 37°C, and then dissociated them into single cells by vortexing. We counted the number of single cells and calculated the number of cells in a single spheroid.

### Immunofluorescence staining

Spheroids were retrieved from each type of microwell and fixed by immersing in 4% paraformaldehyde (PFA) for 30 min at 4°C and then incubating in 0.1% Triton X-100 in PBS for 15 min at room temperature. After washing with PBS containing 0.1% BSA, the spheroids were incubated with 3% BSA at room temperature for 30 min. After aspirating the BSA solution, the spheroids were washed again and incubated overnight at 4°C with mouse anti-albumin antibody (Santa Cruz Biotechnology Inc., Santa Cruz, CA, USA). At this stage, the spheroids were washed with 0.1% BSA and then incubated at 4°C for 90 min with Alexa Fluor 488-conjugated anti-mouse IgG (Invitrogen) secondary antibodies. Other groups of spheroids were prepared as described above and incubated with rabbit anti-cytochrome P450 reductase (Abcam, UK) primary antibodies. These spheroids were washed and incubated with Alexa Fluor 488-conjugated anti-rabbit IgG (Invitrogen) or Alexa Fluor 568-phalloidin (Invitrogen) for F-actin staining, as appropriate. All spheroids were then incubated with DAPI (4',6-diamidino-2-phenylindole) for 5 min at room temperature before being imaged using a confocal microscope (Olympus, Japan).

### Cell viability test

The viability of hepatocytes cultured on the three types of concave microwells was analyzed using a Live/Dead assay (Invitrogen, CA), as described by the manufacturer. 5 mL of primary hepatocyte medium (PHM) containing 2 μL of calcein-AM solution and 5 μL of ethidium homodimer-1 solution were added to each concave microwell, and then incubated at 37°C for 1 h. Stained hepatocytes were analyzed under a fluorescence microscope and confocal microscope (Olympus, Japan).

### Liver function test

Albumin secretion was analyzed using a rat albumin enzyme-linked immunosorbent assay (ELISA) kit (KOMA Biotech, Seoul, Korea) as described by the manufacturer. 300 μL of culture supernatant was collected daily and replaced with 300 μL of fresh DMEM medium for 1 week. Supernatant samples and controls (fresh medium) were added to pre-treated 96-well plates, and absorbance was measured at 450 nm using a microplate reader. Triplicate readings were averaged, and the values obtained by subtracting the converted concentration of the fresh medium from that of the samples were considered to be the concentration of albumin secreted from the spheroids in each microwell.

### Scanning electron microscopy (SEM)

Hepatocyte spheroids formed in circular concave microwells and honeycomb concave microwells were observed under a scanning electron microscope (SEM, JEOL Ltd, Tokyo, Japan). The shape of hepatocyte spheroids was analyzed by fixing the spheroids with 2.5% glutaraldehyde in deionized water (DW) for 1 h, and then washing with DW. In a secondary fixation step, the spheroids were immersed in 1% osmium tetroxide in DW for 1 h and dehydrated using a graded ethanol series (25%, 50%, 75%, 95%, and 100%). After dehydration, the spheroids were washed in tert-butanol three times for 30 min each at room temperature and frozen at −70°C. Hepatocyte spheroids were freeze-dried until the tert-butanol evaporated, and were then mounted onto a specimen stub with graphite paste, coated with palladium alloy, and observed with an SEM.

## Results

### Construction of the jig system and fabrication of honeycomb concave microwell array chip

**Fig 2:**
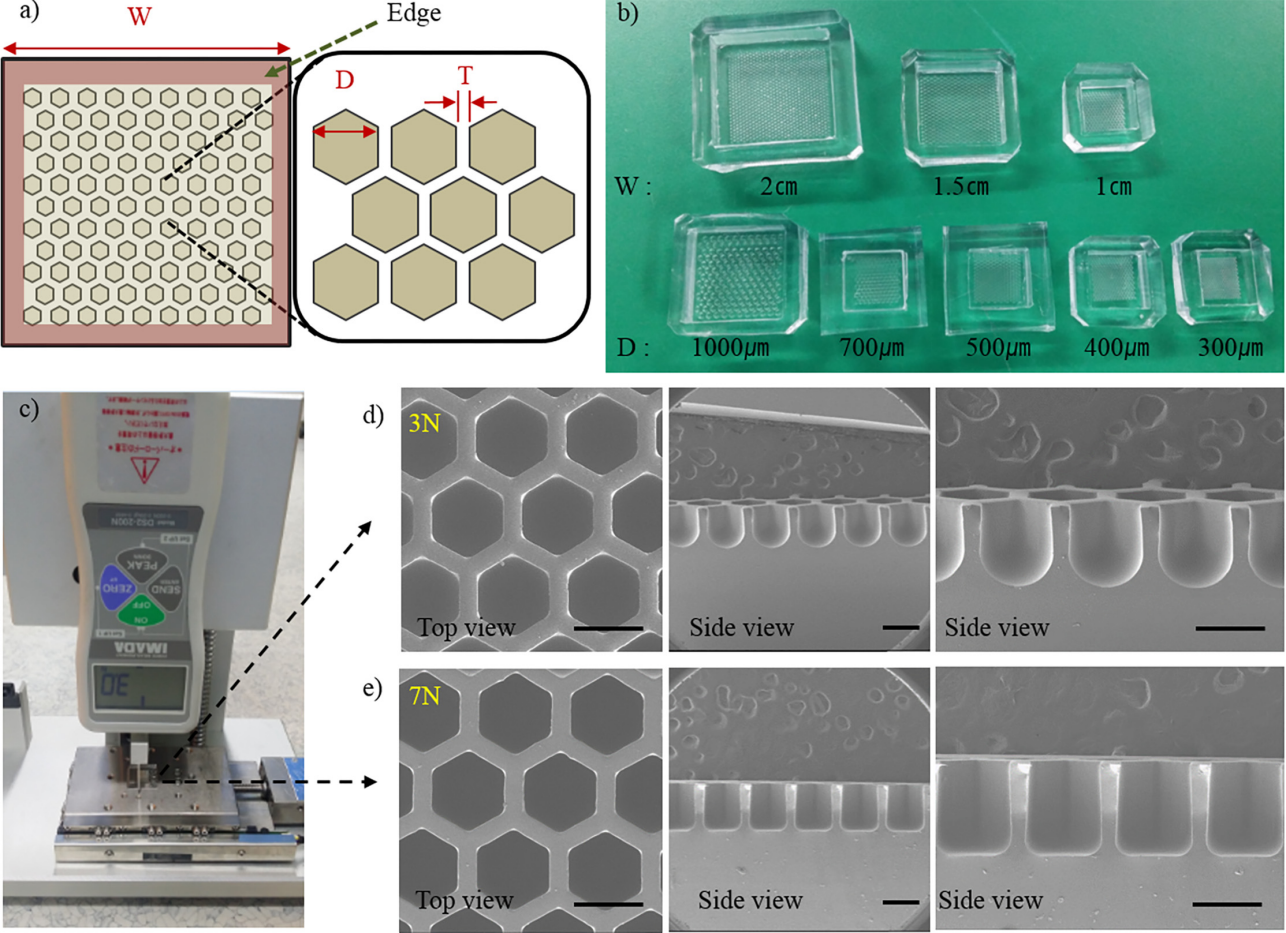
Honeycomb concave microwell and custom-made jig system. (a) Schematic diagram of a honeycomb concave microwell array. W: width; D: diameter; T: wall thickness. (b) A picture of various sizes of honeycomb concave microwell chambers. (c) Pictures of the jig system pressing a chip. Scanning electron microscope (SEM) images of wells formed at a force of (d) 3 N and (e) 7 N showing top views and cross-sectional side views of microwells. Scale bars: 500 μm.

**Fig 3:**
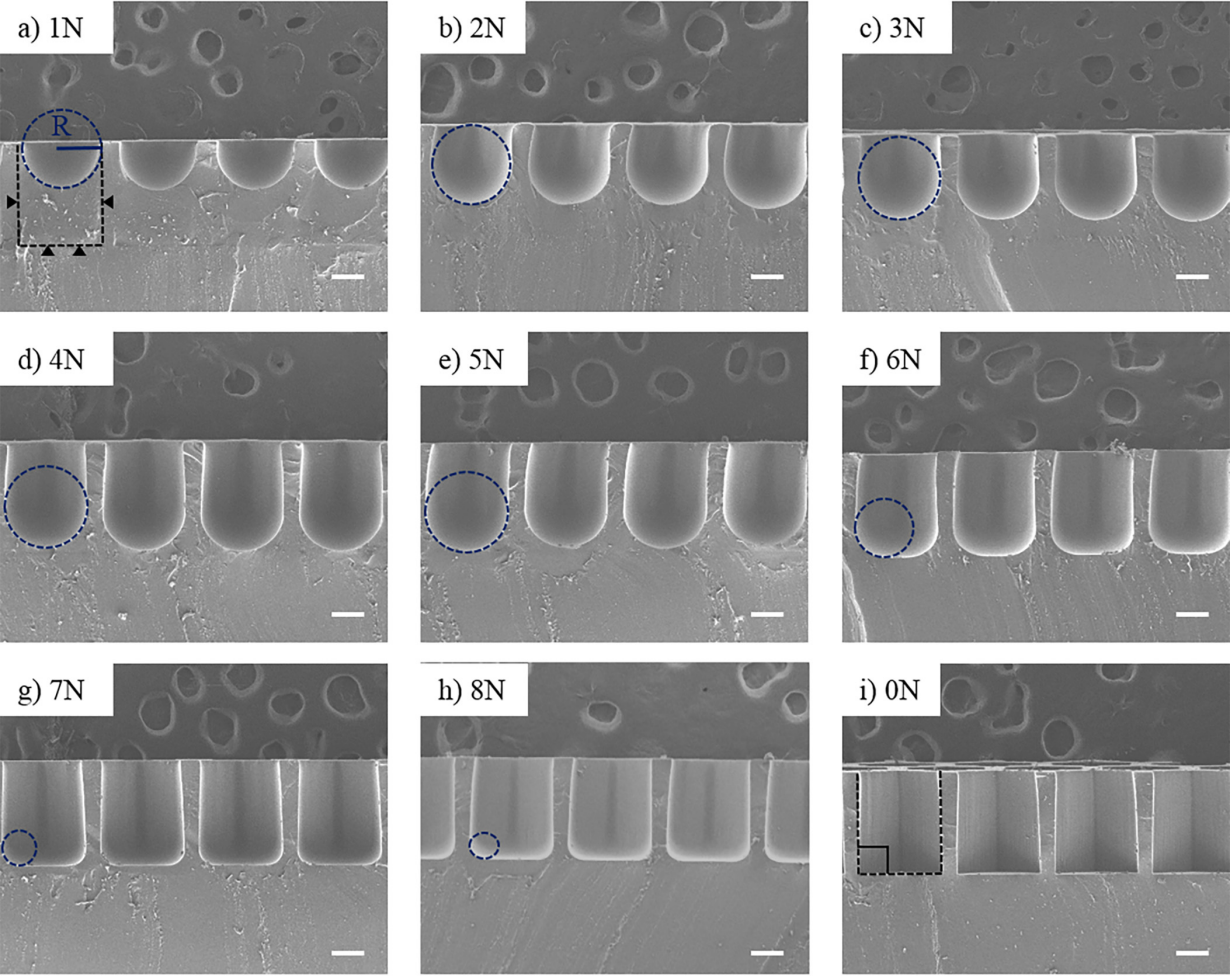
Cross-sectional scanning electron microscopy (SEM) images of honeycomb concave microwell. Curvatures of honeycomb concave microwells formed by applying various forces to the PDMS prepolymer. The black dotted lines and black triangles show the original microwell before pouring the PDMS prepolymer. The blue-dotted circle depicts the radius of the honeycomb concave microwell. Scale bars: 200 μm.

**Fig 4:**
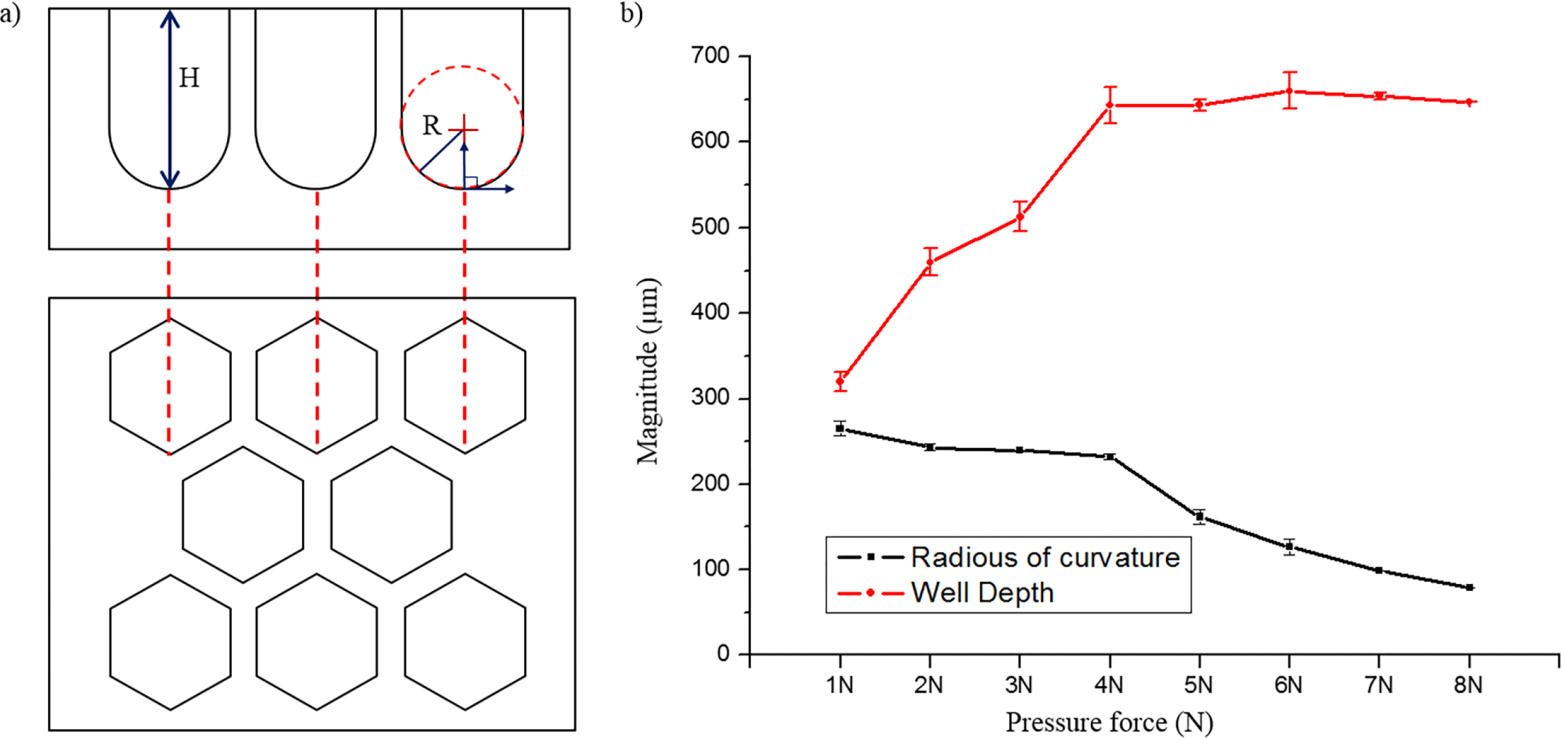
Depth and curvature of honeycomb concave microwells formed by applying various forces to the PDMS prepolymer. (a) Schematic depiction of side and top views of honeycomb concave microwells. (b) Relationship between well depth and radius of curvature of honeycomb concave microwells.

Surface tension-mediated honeycomb concave microwell arrays were successfully fabricated using a custom-made jig system. Fig 2A shows a schematic diagram of the honeycomb concave microwell array. Three different sizes of honeycomb concave microwell chambers (10×10 mm^2^, 15×15 mm^2^, and 20×20 mm^2^) and wells with various diameters (300–1000 μm) were fabricated by raking out the PDMS prepolymer, as shown in Fig 2B. SEM images of top view and side view shows concave bottom of honeycomb concave microwell (S1 Fig). During the raking step, we used a custom-made jig system to fabricate homogeneous and reproducible concave microwells. The depth of the microwells and the radius of curvature of the microwells were determined by the intensity of the pre-set applied force, as shown in Fig 2C and 2D. As the applied force grew higher, the depth and radius of curvature decreased. Force displayed on force meter was changed during raking out procedure as the flat area changed due to the microwell. Force varication during raking out step was analyzed (S2 Fig). A quantitative analysis of the shape of the bottom of the well at different forces was carried out. As shown in the SEM images of the cross-sections of honeycomb concave microwells (Fig 3), the curvature of the surface tension-mediated concave shapes depended on the applied force: as the applied force became higher, the radius of curvature decreased. The depth of concave microwells was also determined by the applied force. ‘0 N’ in Fig 3I indicates a honeycomb microwell fabricated using a standard softlithography method, where the bottom angle is perpendicular to the wall of the microwell. Measurements of the change in height (‘H’) and curvature (‘R’) according to the applied force (defined in Fig 4A) are plotted in Fig 4B, which shows that, as force increases from 1 N to 4 N, the depth of the concave microwell increases while a similar curvature is maintained. At an applied force greater than 5 N, the depth of the well was saturated, though the curvature continued to change.

### Hepatocyte spheroids cultured in concave microwells

**Fig 5:**
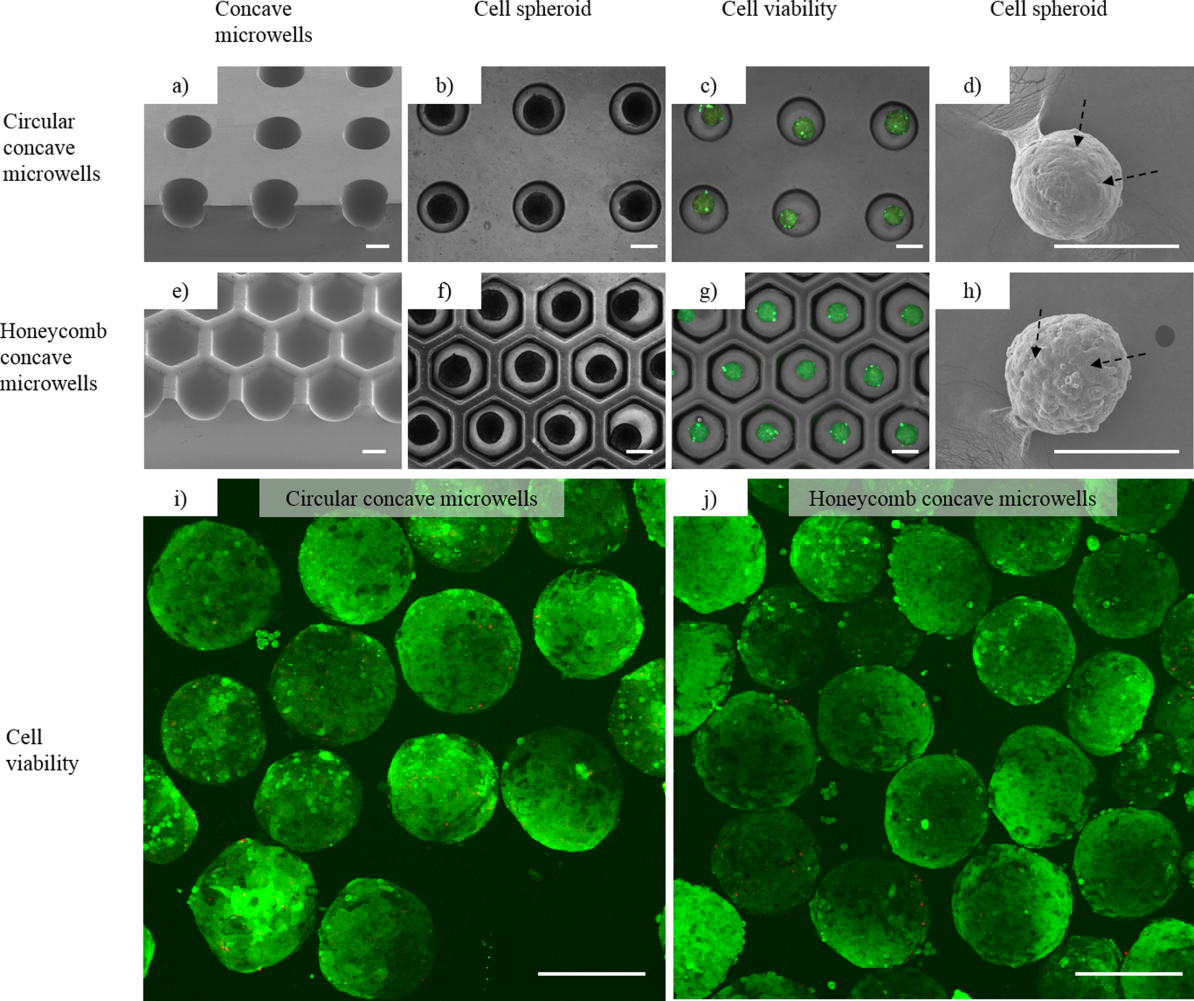
Hepatocytes cultured in (1) circular concave microwells and (2) honeycomb concave microwells. (a, e) Scanning electron microscopy (SEM) images showing the smooth bottom profile of concave microwells 1 and 2. (b, f) Images of hepatocyte spheroids formed in concave microwells 1 and 2. (c, g) Merged images of Calcein AM- and Ethidium homodimer-1-stained spheroids. Calcein AM-stained hepatocyte spheroids (green) cultured for 3 days showing cell viability in concave microwells 1 and 2. Ethidium homodimer-1 fluorescence images (red), which indicate dead cells, are weakly appeared on the images. Scale bars: 500 μm. (d, h) SEM images of spheroids showing formation of tight cell-to-cell junctions in spheroids (black arrows) formed in concave microwells 1 and 2. Confocal microscopic images of cell viability in circular concave microwells (i) and honeycomb concave microwells (j). Scale bars: 250 μm.

To produce homogeneous hepatocyte spheroids, we used two types of chips: circular concave microwells [25, 38] and honeycomb concave microwells (Fig 5A and 5E). Hepatocytes were docked into each microwell and allowed to settle for 5 min after seeding. Within 1 to 2 days after seeding, hepatocytes started to self-aggregate and form spheroids in each concave microwell. Hepatocyte spheroids in the two different chips had stabilized by day 3 (Fig 5B and 5F). Cell viability tests using fluorescence microscope on day 3 showed that more than 95% of the hepatocytes in these groups were viable (Fig 5C and 5G) in concave PDMS microwells, as measured by image analysis using Image J (http://rsbweb.nih.gov/ij/). Fig 5I and 5J also shows more than 95% viability of hepatocytes spheroids measured by confocal microscope after retrieving process from concave microwells. SEM images also showed that hepatocyte spheroids aggregated tightly in each concave microwell (Fig 5D and 5H). These results demonstrate that spheroids form well in both circular concave microwells and honeycomb concave microwells.

### Spheroid size distribution

**Fig 6:**
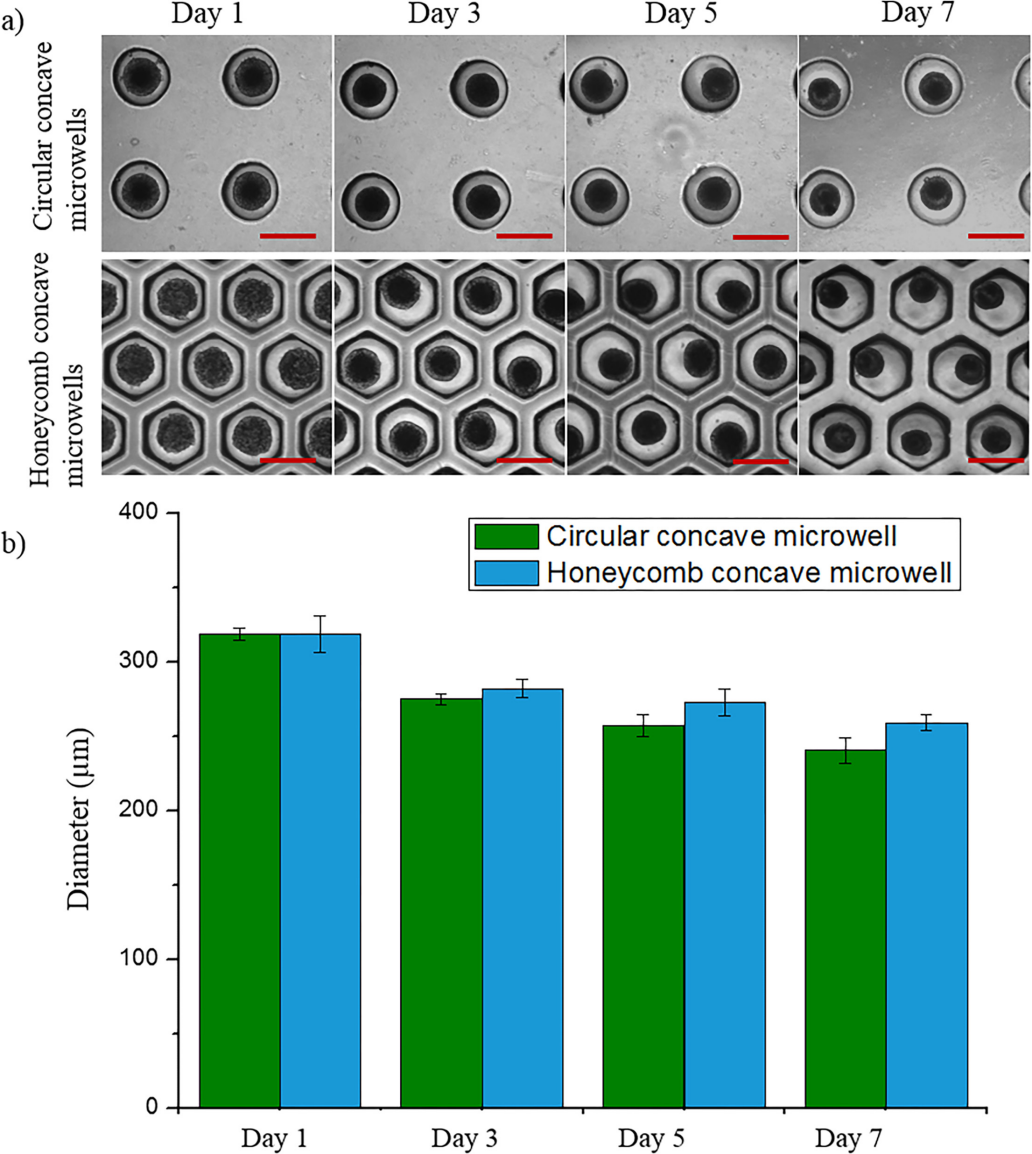
Evaluation of hepatocyte spheroids. (a) Spheroid formation in two types of concave microwells. Scale bars: 500 μm. (b) Size distribution of hepatocyte spheroids in circular and honeycomb concave microwells.

To determine the effect of each culture condition on the size of the spheroids, we analyzed the diameters of hepatocyte spheroids cultured for 7 days using Image J software (Fig 6). The size of spheroids in each microwell decreased through days 1 to 7. After culturing for 1 day in each 500 μm microwell, the average diameters of each spheroid were similar by virtue of the fact that each well had the same radius of curvature and diameter. Spheroids in the honeycomb concave microwells were larger than those in the circular concave microwells because more cells were docked within the concave microwells during the cell-seeding and washing steps. We counted the number of cells in each spheroid on day 7. The number of cells per spheroid in the circular concave microwell and honeycomb concave microwell were 2.3 ± 0.6 × 10^2^ and 3.4 ± 0.2 × 10^2^, respectively. These results show that cells in honeycomb concave microwells were more effectively seeded than those in circular concave microwells, reflecting the fact that the area of a hexagon is 10.3% greater than that of the inscribed circle.

### Comparison of cell loss

In order to investigate the relationship between the shape of the microwell and the corresponding cell loss, we performed a cell-loss test. Cells in the external region of each microwell were gathered by pipetting, and we then counted the number of retrieved cells. A high percentage (65.3%) of cells was lost in circular concave microwells because these arrays have a smaller number of microwells and a larger amount of dead space (Table 1). The percentage of cells lost was lower for dense concave wells (26%) owing to a smaller dead space. As predicted, the percentage of cells lost was much lower for honeycomb concave wells (22%) owing to the smaller amount of dead space. Cells seeded on the top of thin walls apparently fell into the honeycomb concave microwells.

**Table 1:** Comparison of cell loss after washing in concave microwells.

Well types	Number of wells	Flat area in concave microwell chamber (%)	Cell loss (%)
Sparse concave microwell	100	80.4	65.3
Dense concave microwell	196	61.5	26
Honeycomb concave microwell	196	57.6	22

### Liver-specific function test

**Fig 7:**
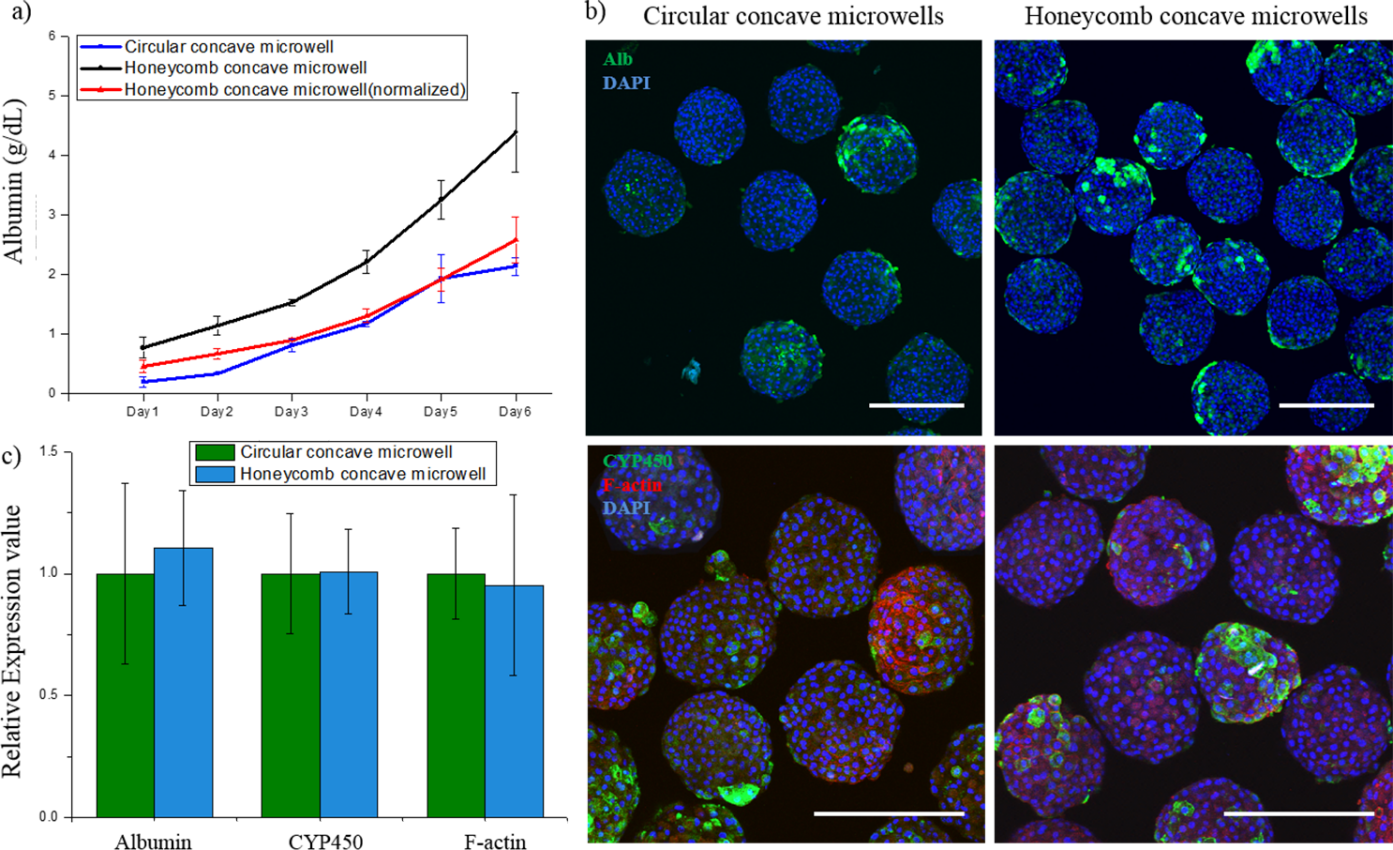
Hepatocyte spheroids in honeycomb concave microwells exhibit greater liver-specific function than those in circular concave microwells. (a) Analysis of metabolic functions of spheroids in the two types of microwells, as measured by the secretion of albumin. (b) Immunostaining of hepatocyte spheroids for albumin (primary hepatocytes; green in first row), DAPI (nuclei; blue), cytochrome P450 activity (CYP450; green in second row) and microfilaments (F-actin; red). (c) Quantified analysis of hepatocytes function test. Scale bars: 300 μm.

To analyze the liver-specific functions of hepatocyte spheroids in each type of microwell, we performed immunostaining for albumin, CYP450, and F-actin, and assessed the albumin secretion using ELISA. The liver-specific function test was performed for each concave microwell. As shown in Fig 7A, the total albumin concentration in the honeycomb concave microwell groups (black line) was 2.1 times higher than that in the circular group (blue line) after 1 week of cultivation. The normalized albumin concentration in the honeycomb concave microwell group (red line) was plotted for direct comparison with the circular group. As shown in the immunofluorescent images (Fig 7B), the albumin expression by spheroids in the honeycomb group was much higher than that in the circular group because the total number of retained cells was higher. The metabolic activities of hepatocytes and cell-cell junctions in spheroids were similar in all two groups, as evidenced by the fluorescence images that show the expression of CYP450 reductase and F-actin. Quantified data of CYP450, F-actin and albumin was analyzed in Fig 7C.

## Discussion

We fabricated a deep honeycomb concave microwell array by exploiting the viscoelastic properties and surface tension of the PDMS prepolymer. Using a custom-developed jig system, we demonstrated the successful and reproducible formation of uniformly sized concave microwells. In our previous study, we fabricated concave microwells for the production of embryoid bodies by raking out the PDMS prepolymer using glass slides [38]. Although this method is cost-effective and requires minimal labor compared with conventional micro-fabrication methods–such as deep reactive ion etching (DRIE) [46], cylindrical microchannel formation using a water mold [47], and ice droplet-based microcavity formation [31]–it still has a limited ability to reproducibly fabricate uniform, controllably shaped concave structures in a dense manner. To address this challenge, we developed a jig system composed of a ball screw, LM guide block, digital vernier caliper, digital force gauge, and motorized linear stage. Regardless of the well shape and distance between wells, the developed jig system successfully created concave microwells whose depths and curvatures were determined by the magnitude of the force applied during the process of raking the PDMS prepolymer. The raking speed used to form a meniscus was 5 mm/s. Homogeneous meniscus formation was observed under a raking speed of 10 mm/s. This jig system enabled the fabrication of uniformly sized concave microwells with thin walls and with a high reproducibility. If the wall thickness was smaller than 100 μm, it became difficult to separate the PDMS from the master mold without damage. However, the proposed method enabled the construction of a honeycomb concave structure with thin, high walls (100 μm in width, 650 μm in height). Various sizes of the honeycomb concave microwell were able to be fabricated, as shown in Fig 2B. SEM top-view and side-view images of the honeycomb concave microwell are shown in S1 Fig.

Upon using the concave honeycomb wells, uniformly shaped and sized spheroids were successfully fabricated from seeded rat hepatocytes. Cell loss during cell seeding was minimized, and the number of spheroids within a certain area was maximized by employing a compact honeycomb structure. A microwell depth of 650 μm was sufficiently deep to avoid the escape of spheroids from their original position during media change. As expected, cell loss during cell seeding in the circular concave microwell was high (>65%), as shown in Table 1. Although these lost cells can be reused, the retrieval process is inefficient, laborious, and time-consuming. By way of contrast, cell loss in the honeycomb-shaped concave microwell was reduced to 22%. Most cell loss in the honeycomb concave wells was attributable to the flat areas at the edge of the microwells (defined in Fig 2A), and we expect that this cell loss could be decreased by optimizing the shape of the edge. Cell loss on the top of the wall was rarely observed.

The function of hepatocyte spheroids was evaluated by assaying albumin secretion over 6 days and immunostaining for CYP450 reductase as an indicator of enzymatic activity. All spheroids in the two types of microwells secreted albumin continuously and showed a similar degree of enzymatic activity. However, the honeycomb concave microwells were highly effective in producing large numbers of functional spheroids upon using the same seeding and culturing procedures, as evidenced by the results of cell-loss tests and albumin ELISAs. On the basis of these results, we conclude that our honeycomb concave microwell model could provide an appropriate platform for producing uniformly sized spheroids with minimal cell loss, as well as high spheroid density and viability.

## Conclusion

In this paper, we have developed a simple and cost-effective method for fabricating PDMS-based honeycomb concave microwell arrays using a jig system that enables control over the radius of curvature and well depth, thereby producing uniformly sized hepatocyte spheroids. The densely packed honeycomb concave microwells greatly increased the cell seeding efficiency. This system could be used for other types of agglomerate cells to form uniform-sized 3D spheroids with minimum cell loss. Additionally, the proposed method for spheroid production has extensive potential applications in tissue engineering, organ regeneration, the study of organs and diseases, and the screening of drug and toxicity.

## Supporting Information

S1 Fig Scanning electron microscopy (SEM) images of honeycomb concave microwells. Top view and side view of (a) 300, (b) 400, (c) 500, (d) 700, and (e) 1000 μm diameter wells. Side-view images of different chip sizes: (f) 10×10 mm^2^, (g) 15×15 mm^2^, and (h) 20×20 mm^2^. Scale bars: 300 μm.
(TIF)

S2 Fig Force varication during raking out procedure (10×10 mm^2^ size chip). (TIF)
