# Supplementary figures and images for: Reproducible Construction of Surface Tension-Mediated Honeycomb Concave Microwell Arrays for Engineering of 3D Microtissues with Minimal Cell Loss
Source: PLoS One. 2016 Aug 11;11(8):e0161026. doi: 10.1371/journal.pone.0161026 (PMC4981302; doi:10.1371/journal.pone.0161026)

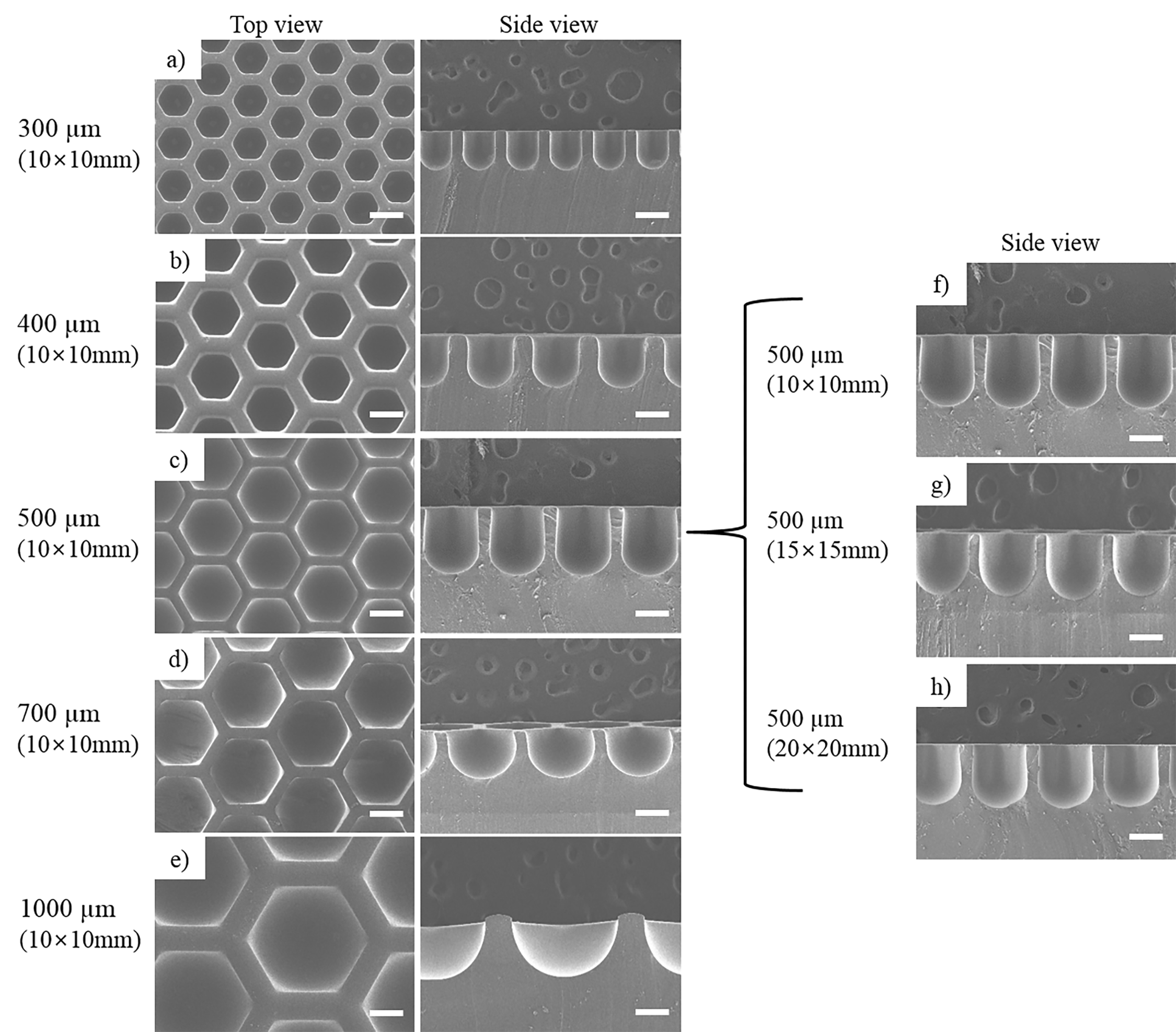

Supplement: S1 Fig — Top view and side view of (a) 300, (b) 400, (c) 500, (d) 700, and (e) 1000 μm diameter wells. Side-view images of different chip sizes: (f) 10×10 mm2, (g) 15×15 mm2, and (h) 20×20 mm2. Scale bars: 300 μm. (TIF) [file pone.0161026.s001.tif]

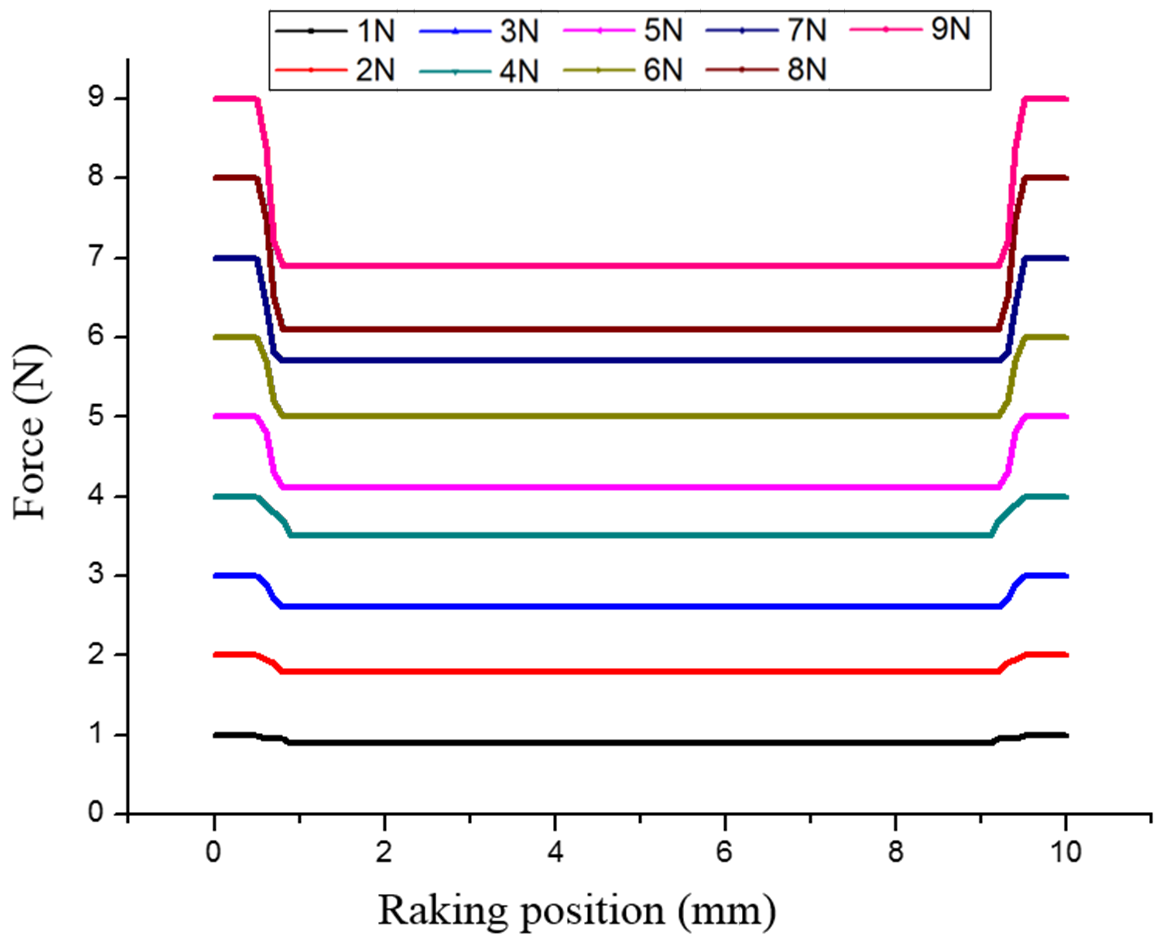

Supplement: S2 Fig — (TIF) [file pone.0161026.s002.tif]
